# Supplementary material for: Investigation of SARS-CoV-2 infection in dogs and cats of humans diagnosed with COVID-19 in Rio de Janeiro, Brazil
Source: PLoS One. 2021 Apr 28;16(4):e0250853. doi: 10.1371/journal.pone.0250853 (PMC8081175; doi:10.1371/journal.pone.0250853)
Supplement: S1 Fig — (DOCX) [file pone.0250853.s001.docx]

**S1 Fig. Flow diagram of the study**

Assessed for eligibility (n = 102)

(Men = 42 (41.2%), Women = 60 (58.8%))

## Screening

## Enrollment

Enrollment (n = 21)

Men: 8 (38.1%), median age: 37.0 (IQR; 27.3, 44.5)

Women: 13 (61.9%), median age: 40.0 (IQR; 34.5, 55.5)

39 companion pets: 29 dogs and 10 cats

## Follow-Up

19 human patients completed the study

27 dogs and 7 cats completed the study

Reasons for discontinuation (n= 3)

Lost to follow-up, missed allowable visit window =3

Excluded (n= 81)

34 (42%) Men, median age: 37.5 (IQR; 31.5, 54.3)

47 (58%) Women, median age: 38.0 (IQR; 30.0, 51.0)

Reasons:

-Without companion pets: 62 (76.5%)

-No reported reason: 9 (11.1%)

-Met an exclusion criterion (<6 months or > 10 years for sedation): 2 (2.5%)

-Did not answer veterinarian’s phone calls: 2 (2.5%)

-Owner lived in an area of difficult access: 2 (2.5%)

-Owner referred that the animal is extremely aggressive: 1 (1.25%)

-Owner did not attend the first scheduled visit: 1 (1.25%)

-Owner stayed out of the home during the entire COVID-19 disease course far from the pet: 1 (1.25%)

-Owner did not allow pet sample collection: 1 (1.25%)
