# Supplementary material for: Investigation of SARS-CoV-2 infection in dogs and cats of humans diagnosed with COVID-19 in Rio de Janeiro, Brazil
Source: PLoS One. 2021 Apr 28;16(4):e0250853. doi: 10.1371/journal.pone.0250853 (PMC8081175; doi:10.1371/journal.pone.0250853)
Supplement: S1 Table — (DOCX) [file pone.0250853.s002.docx]

**S1 Table. Primers used to amplify fragments of the genome of SARS-CoV-2 in the present study.**

| **Primer** | **Sequence 5'-3'** | **Tm** | **Position according to the reference strain*** | | |
| --- | --- | --- | --- | --- | --- |
| hCoV-19_54F | TGCAAAGAATAGAGCTCGCACC | 61,3 | 15086 | 15108 |  |
| hCoV-19_54R | CGCCACACATGACCATTTCACT | 61,7 | 15451 | 15429 |  |
| hCoV-19_55F | TGCTCGCAAACATACAACGTGT | 61,6 | 15353 | 15375 |  |
| hCoV-19_55R | AGCCACTAGACCTTGAGATGCA | 61,2 | 15771 | 15749 |  |
| hCoV-19_56F | TTTGTGAATGAGTTTTACGCATATTTGC | 60,4 | 15660 | 15688 |  |
| hCoV-19_56R | AGCTAAAGACACGAACCGTTCA | 60,2 | 16029 | 16007 |  |
| hCoV-19_88F | ACCACAAATCATTACTACAGACAACACA | 61,3 | 24894 | 24922 |  |
| hCoV-19_88R | GCAGCAGGATCCACAAGAACAA | 61,4 | 25324 | 25302 |  |
| hCoV-19_89F | CTAGGTTTTATAGCTGGCTTGATTGC | 60,5 | 25213 | 25239 |  |
| hCoV-19_89R | TGGAGAGTGCTAGTTGCCATCT | 61,2 | 25615 | 25593 |  |
| hCoV-19_90F | TTCGGATGGCTTATTGTTGGCG | 61,8 | 25518 | 25540 |  |
| hCoV-19_90R | GACTTGTTGTGCCATCACCTGA | 61,1 | 25924 | 25902 |  |

***** hCoV-19/Wuhan/WIV04/2019 (WIV04) (EPI_ISL_402124)
