# Supplementary material for: Investigation of SARS-CoV-2 infection in dogs and cats of humans diagnosed with COVID-19 in Rio de Janeiro, Brazil
Source: PLoS One. 2021 Apr 28;16(4):e0250853. doi: 10.1371/journal.pone.0250853 (PMC8081175; doi:10.1371/journal.pone.0250853)
Supplement: S2 Table — (DOCX) [file pone.0250853.s003.docx]

**S2 Table. Baseline characteristics of 21 index human study participants between May 2^nd^, 2020 and October 7^th^, 2020 (metropolitan region of the state of Rio de Janeiro, Brazil).**

| **Characteristics** | **n (%)** |
| --- | --- |
| **Sex, n (%)**  Men  Women | 8 (38.1)  13 (61.9) |
| **Age, years, median (IQR)** | 39.9 (32.7- 48.9) |
| **Race/ethnicity, n (%)**  White  Black  Mixed | 14 (66.6)  1 (4.8)  6 (28.6) |
| **Marital Status, n (%)**       Single  Married  Living with a partner  Separated or Divorced  Widowed | 10 (47.6)  8 (38.1)  1 (4.8)  1 (4.8)  1 (4.8) |
| **Highest formal educational attendance, n (%)**  University/post-graduation       High school/technical college       Lower secondary  Primary | 15 (71.4)  4 (19.0)  1 (4.8)  1 (4.8) |
| **Self-reported history of chronic diseases, yes, n (%)** |  |
| Rhinitis  Hypertension  Obesity  Joint Disease  Chronic obstructive pulmonary disease  History of stroke  HIV infection  Coronaropathy  Anemia  Asthma | 7 (33.3)  7 (33.3)  3 (14.3)  2 (9.5)  2 (9.5)  2 (9.5)  1 (4.8)  1 (4.8)  1 (4.8)  1 (4.8) |
| **Influenza virus immunization, last 12 months, yes, n (%)** | 14(66.7) |
| **History of allergies, yes, n (%)** | 10 (47.6) |
| **Current smoker, yes, n (%)** | 1 (4.8) |
| **Signs and symptoms, yes, n (%)**  Hoarseness  Prostration  Cough  Headache  Earache  Loss of taste  Loss of smell  Myalgia  Chills  Anorexia  Diarrhea  Nasal congestion  Oropharyngeal pain  Dyspnea  Coryza  Fever  Arthralgia  Xerostomia  Retro-orbital pain  Nausea  Eye burning  Sweating  Mouth ulcers  Tingling  Abdominal pain  Photophobia  Vomiting | 17 (81.0)  17 (81.0)  15 (71.4)  14 (66.7)  14 (66.7)  12 (57.1)  11 (52.4)  11 (52.4)  9 (42.9)  9 (42.9)  9 (42.9)  8 (38.1)  8 (38.1)  7 (33.3)  7 (33.3)  6 (28.6)  4 (19.0)  4 (19.0)  4 (19.0)  3 (14.3)  3 (14.3)  3 (14.3)  2 (9.5)  2 (9.5)  1 (4.8)  1 (4.8)  w1 (4.8) |

IQR: Interquartile Range.
