# Supplementary material for: Investigation of SARS-CoV-2 infection in dogs and cats of humans diagnosed with COVID-19 in Rio de Janeiro, Brazil
Source: PLoS One. 2021 Apr 28;16(4):e0250853. doi: 10.1371/journal.pone.0250853 (PMC8081175; doi:10.1371/journal.pone.0250853)
Supplement: S3 Table — (DOCX) [file pone.0250853.s004.docx]

**S3 Table. The number of cats and dogs and samples tested for RT-PCR for the detection of SARS-CoV-2 in the study, between May 2^nd^, 2020 and October 7^th^, 2020 (metropolitan region of the state of Rio de Janeiro, Brazil).**

|  | **Sampling I** | | **Sampling II** | | **Sampling III** | |  |
| --- | --- | --- | --- | --- | --- | --- | --- |
| **Species** | **NP/OP swab** | **Rectal swab** | **NP/OP swab** | **Rectal swab** | **NP/OP swab** | **Rectal swab** | **Total** |
| **Dogs (n=29)** | 29 | 29 | 28 | 28 | 27 | 27 | 168 |
| **Cats**  **(n=10)** | 10 | 10 | 5 | 5 | 7 | 7 | 44 |
| **Total** | 39 | 39 | 33 | 33 | 34 | 34 | 212 |

n= number of animals.
