# Supplementary material for: Investigation of SARS-CoV-2 infection in dogs and cats of humans diagnosed with COVID-19 in Rio de Janeiro, Brazil
Source: PLoS One. 2021 Apr 28;16(4):e0250853. doi: 10.1371/journal.pone.0250853 (PMC8081175; doi:10.1371/journal.pone.0250853)
Supplement: Attachment — Submitted filename: Response to Reviewers_15.03.2021.docx [file pone.0250853.s005.docx]

Manuscript number: PONE-D-21-02395

Title: Investigation of SARS-CoV-2 Infection in Dogs and Cats of Humans Diagnosed with COVID-19 in Rio de Janeiro, Brazil

PLOS ONE

**Corresponding author:** Guilherme Calvet

| REVIEWER COMMENTS | RESPONSE | **Reference page**  *(Where the change now appears in the newly revised manuscript)* |
| --- | --- | --- |
| **EDITOR** | |  |
| If there are ethical or legal restrictions on sharing a de-identified data set, please explain them in detail (e.g., data contain potentially identifying or sensitive patient information) and who has imposed them (e.g., an ethics committee). Please also provide contact information for a data access committee, ethics committee, or other institutional body to which data requests may be sent.  If there are no restrictions, please upload the minimal anonymized data set necessary to replicate your study findings as either Supporting Information files or to a stable, public repository and provide us with the relevant URLs, DOIs, or accession numbers. Please see http://www.bmj.com/content/340/bmj.c181.long for guidelines on how to de-identify and prepare clinical data for publication. For a list of acceptable repositories, please see http://journals.plos.org/plosone/s/data-availability#loc-recommended-repositories. | Yes. As we collected several personally identifiers and sensitive data from the study participants, we ask the editors to consider the following statement to be written in the manuscript, if accepted for publication:  **Data Availability:** Data underlying the study cannot be made publicly available due to ethical concerns, as data contain several personally identifiable information. Data are available from Oswaldo Cruz Foundation for researchers who meet the criteria for access to confidential data. Contact information: Institutional Ethics and Research Committee of the Evandro Chagas National Institute of Infectious Diseases, email: [cep@ini.fiocruz.br](mailto:cep@ini.fiocruz.br) or Guilherme Amaral Calvet; email: [guilherme.calvet@ini.fiocruz.br](mailto:guilherme.calvet@ini.fiocruz.br) | Not applicable |
| We note that you have included the phrase “data not shown” in your manuscript. Unfortunately, this does not meet our data sharing requirements. PLOS does not permit references to inaccessible data. We require that authors provide all relevant data within the paper, Supporting Information files, or in an acceptable, public repository. Please add a citation to support this phrase or upload the data that corresponds with these findings to a stable repository (such as Figshare or Dryad) and provide and URLs, DOIs, or accession numbers that may be used to access these data. Or, if the data are not a core part of the research being presented in your study, we ask that you remove the phrase that refers to these data. | This is an important point, thank you. We agree with the editor and have deleted this sentence because the data are not a core part of the present research being presented. | Not applicable |
| **REVIEWER 1** | |  |
| The manuscript has stated objectives:  1) To assess the exposure, infection, and persistence of SARS-CoV-2 in nasopharyngeal and oropharyngeal secretions (nasal and oral swabs) and feces (rectal swab) of dogs and cats living in households with a COVID-19 human case. 2) To investigate clinical and laboratory alterations (complete blood count and serum biochemistry) in pets associated with the SARS-CoV-2 infection. 3) To evaluate risk factors associated with SARS-CoV-2 infection in pets.  However, the objective 2 does not appear to be pertinent to the context of the manuscript and its results were not really discussed. I recommend the second objective and related parts of the manuscript be removed. | We agree with the reviewer and remove the objective 2 from the text. But we have decided to keep the results in the article. So far, few published articles provide complete blood count and biochemistry results. All pets performed these tests at three different times. We believe that maintaining this data adds information to the study. | Not applicable |
| 144, 210, 212, 414 etc.: “… human’s samples” should be “… human samples” | Thank you for your pertinent suggestion. We have changed human’s samples” to “human samples”. | Lines: 139, 193, 195. |
| 145-147: “All VTM samples had viral RNA extracted manually by the QIAamp Viral RNA Mini kit (Qiagen), or automatedly by Perkin-Elmer Chemagic machine/chemistry…” should be re-phrased because QIAamp Viral RNA kit actually extracts both DNA and RNA, that is total nucleic acids (no DNase treatment). If Perkin-Elmer Chemagic does not include DNase than it would be appropriate to say: “Nucleic acids were extracted from VTM samples by QIAamp Viral RNA Mini kit (Qiagen), or by Chemagic instrument (Perkin-Elmer)…”  Also please specify the instrument model – was it Chemagic Prime, Chemagic 360 or Chemagic Prepito? | Thank you for this relevant recommendation. We agree with the reviewer’s points and have corrected the text as suggested.  New sentence: Nucleic acids were extracted from VTM samples by QIAamp Viral RNA Mini kit (Qiagen), or by Chemagic 360 instrument (Perkin-Elmer). | Lines: 140-141. |
| 150: “Amplifications were conducted …” should be changed to “Reverse transcription and amplification were conducted…” | Thank you for this very appropriate suggestion. We have included “Reverse transcription and” in the sentence. | Lines: 143-144. |
| 213-215: “…tested by a RT-PCR designed to amplify N1 and N2 genes of SARS-CoV-2, as previously described [22]. Pet samples were only considered positive for SARS-CoV-2 when at least two genes were amplified.”  This should be re-phrased because: (i) it does not match the reference which describes detection of RdRp, E and N genes by real-time PCR assays; (ii) SARS-CoV-2 does not have N1 and N2 genes. | We have changed the sentence and included a new reference to support the statement made in the text.  "...tested by a RT-PCR designed to amplify two targets of gene N of SARS-CoV-2, as previously described [23]  23. Centers for Disease Control and Prevention. 2019-Novel coronavirus (2019- nCoV) real-time rRT-PCR panel primers and probes. [Updated June 6 2020]. Available at: https://www.cdc.gov/coronavirus/2019-ncov/lab/rt-pcr-panel-primer-probes.html. Accessed March 12, 2021. | Lines: 195-197. |
| 244-145 “The PRNT is a highly specific serologic test and can be carried out on samples to confirm the presence of neutralizing antibodies to SARS-CoV-2 [24].” Should be re-phrased because stating “to confirm the presence” implies a screening test was done before, therefore it is more appropriate to state” The PRNT is a highly specific serologic test for detection of neutralizing antibodies to SARS-CoV-2 [24].” | We agree with this pertinent comment. We have changed the sentence to: “The PRNT is a highly specific serologic test for detection of neutralizing antibodies to SARS-CoV-2.” | Lines: 227-228. |
| 252: research facility of biosafety level 3 platform of IOC/Fiocruz. – define “IOC” | Thank you for pointing this out. We have changed the sentence to: “**…** all manipulation of infectious SARS-CoV-2 was performed in a multi-user research facility of biosafety level 3 platform of Oswaldo Cruz Institute (IOC)/Fiocruz. | Lines: 232-235. |
| **REVIEWER 2** | |  |
| The material and methods section was extensively written, Please reduce it | Thank you for this recommendation. We have decided to delete some parts of the text and rephrase others to reduce the section. | Not applicable |
| - Have you considered sampling dogs and cat from nearby households with negative SARS-CoV-2 owners , as a mean to add a control element to the study. | Thank you for your comment. But we haven't considered sampling dogs and cats from nearby households with negative SARS-CoV-2 owners. Our multidisciplinary project was developed in partnership with the Acute Febrile Illnesses Laboratory, and the Laboratory of Clinical Research on Dermatozoonoses in Domestic Animals, basing the collections of the animals after the identification of the human clinical cases that are being monitored. | Not applicable |
| - Line 58: replace (infected/exposed) with (positive) - In the entire text replace the word “exposed” and use the world “ infected or positive” because the demarcation between the two words is not sufficiently clear in the current prospective. | Thanks for the comment. We clarify that we used the word "exposed" to designate the presence of only neutralizing antibodies since there was no direct diagnosis with the presence of RNA or viral isolation. The presence of these antibodies indicates only previous exposure to the SARS-CoV-2. But we replace the term "exposed" with "seropositive" in the entire text for a better understanding.  We adopted the use of the term "infected" as defined by The World Organization for Animal Health (OIE) definition criteria for confirmed cases (infection) of SARS-CoV-2 in animals. | Lines: 58, 62, 238, 248, 331, 346, 350, 445, 448, 452, 455, 460, 560, 580. |
| -Line 342: the use of plasma in serological testing is not recommended because of the possibility of nonspecific result. Please discard, if any, serological results based on the use of plasma. | Different biological samples are used to search for neutralizing antibodies, including serum, plasma, and cerebrospinal fluid. All of them undergo inactivation for 56 to 30 minutes to destroy proteins in the complement system that could influence neutralization. Besides, none of the tested plasma samples were seropositive in the study, and therefore there was no unspecific neutralization of the tested plasma samples. We have added the number of serum (n=92) and plasma (n=8) samples tested in the study. | Lines: 324-325. |
| Line 514 this sentence should be removed “It was also experimentally demonstrated that cats can become infected and 515 transmit SARS-CoV-2 to other cats” because of its poor relation to the rest of the paragraph. | Thank you for the insightful comment. We have deleted the sentence. | Not applicable |
| **REVIEWER 3** | |  |
| 1- One of the major concerns is the lack of description and comparison of the obtained sequences from animals to that of the circulating strains in humans at the time of their longitudinal study. It is highly recommended to sequence the full-length genome or at least the full-length S gene sequences of some isolates from animals and compare it to that of human. Such information will help great impacts in improving the quality of this manuscript. Meanwhile, presenting theses sequences and making them publically available is of high demands. | We agree with this study's limitation and that we recognize and describe in lines 527 to 535 (original manuscript). Also, all sequences obtained in the animals sampled in this study are deposited in an open-access database (GISAID), and their access numbers are described in table two. | Not applicable |
| 2- The authors must map in more precise details the sampling schedules from both human and animals. | Thank you for this recommendation. We have detailed sampling schedules. | Lines: 130-132. |
| 3- A detailed description of the outcomes of the viral infection in animals in correlation to human must be presented. | None of the cases of human patients diagnosed with COVID-19 needed hospitalization and the infection resolved itself with no signs of sequelae. Also, there was no need for specific medical care during the follow-up appointments of the animals positive for SARS-CoV-2. No human or animal deaths were observed during the study.  We have included this information in the text. | Lines: 288-291. |
| 4- The statistical analysis in the M&M is long. Please consider shortening this section and use key references instead. | Thank you for your suggestion. We have decided to delete some parts of the statistical analysis section. | Not applicable |
| 5- The discussion section is very long. Some paragraphs are too vague. Please consider shortening theses sections and mainly focusing this section on discussing your findings in comparison to other international groups that have conduced similar studies especially from USA, Asia, and Europe. | Thank you for pointing this out. We agree with the reviewer and delete some sentences in the discussion section. | Not applicable |
| 6- Please consider deleting Objectiv-2 since it is not adding too much to the main theme of this manuscript. | Thank you for your comment. We decided to delete the objective 2 in the introduction section of the manuscript, but we have decided to keep the results in the article as previously mentioned in the response to reviewer #1. | Not applicable |
| 7- Please try to highlight the roles of the One Health concept as an important approach for studying this type of research and its potential roles in the mitigation and control of the emerging and re-emerging diseases. | Thank you for this relevant recommendation. We have included the following paragraph in the discussion section:  “The present study highlights the role of the One Health approach in the mitigation and control of the COVID-19 pandemic [44, 45]. The integrated surveillance infrastructure of the Evandro Chagas National Institute of Infectious Diseases and the Regional Reference Laboratory in the Americas for Coronavirus was able to timely monitor and detect the occurrence of SARS-CoV-2 infection in both humans and animals using a multi-professional approach, including veterinarians, physicians, other health professionals, and a statistician The results of our study have been provided to The Brazilian Ministry of Agriculture, Livestock and Food Supply and can contribute to the investigation of animal SARS-CoV-2 hot spots. | Lines: 515-523. |
